# Gleanings from Our Exchanges

**Published:** 1872-08-15

**Authors:** 


### Gleanings from Our Exchanges.

### BLINDNESS AND DEAFNESS IN CONSEQUENCE OF EPIDEMIC CEREBRO-SPINAL MENINGITIS.

BY H. KNAPP, M. D.

During the last three months and a half there have come under my care forty-one cases of blindness or deafness, the consequence of cerebro-spinal meningitis, which has for more than six months assumed an epidemic character in this part of the country. Among the forty-one patients thirty-one were deaf on both sides, eight were blind with one eye, mostly on left, one was blind with both eyes, and one was deaf on both sides and blind on one. In all cases the diagnosis of cerebro-spinal meningitis could be well established. The symptoms were—mostly a sudden attack of headache, increase of temperature, vomiting, convulsions with predominant opisthotonos, unconsciousness, deliria, staggering gait with a tendency to fall sideways, lasting for weeks and months after the recovery. Among the complications I may mention inflammations of the joints in some cases.

The affection of *the eye* begins usually in the first weeks of the general disease, with the following symptoms: Circumcorneal injection, discolored iris, ragged pupil, fundus occuli dull, its details not recognizable, or the fundus yielding only a somber red color, or appears black. Hypopyon and yellowish exudation plugging the pupil are not infrequent. The cornea, in some rare cases, becomes ulcerous; in others, the conjunctiva and lids are œdematous and very red, the eyeball protrudes, and exceptionally bursts, suppurates and shrinks. Ordinarily, the injection of the conjunctiva subsides, the cornea clears up, the hypopyon and exudation in the pupil disappear, and the eyeball assumes a strikingly peculiar and characteristic appearance, which I have only seen in cerebro-spinal meningitis, puerperal fever, and very seldom in typhoid and typhus fevers. The iris is dull and bulges forward like a cone. Its periphery, however, is usually drawn backward. The pupil is rather narrow, ragged and immovable. Through the transparent lens, which has advanced with the iris, a dull white surface is visible in the vitreous chamber. The eyeball is commonly softer and smaller than natural. Sight is completely and irrevocably lost. Later, the crystalline becomes cataractous. The eyeball will remain smaller and softer, squint outward, but never give rise to other inflammations, or sympathetic affection of the other eye. I supply these facts from my observations of the epidemic of cerebro-spinal meningitis which reigned in the upper valley of the Rhine eight years ago. The eye-affection has been mistaken for medullary cancer (glioma) of the retina, but may be distinguished from it by its acute development in combination with the general disease, the peculiar protrusion of the center of the iris and its retraction at the periphery, the dull white reflexion from behind the pupil, and the diminution of size and tension of the globe.

The nature of the eye-affection is purulent choroiditis, probably metastatic. There have been other changes of the eye observed in cerebro-spinal meningitis, principally hyperæmia and inflammation in and around the optic disk. They are rare and not specifically dependent upon this form of meningitis, but on hyperæmia, exudation, and proliferation in the cranial cavity in general.

The *ear-affection* in cerebro-spinal meningitis does not show symptoms so peculiar as the eye-affection. In the early stage hyperæmia of the middle ear is commonly present, the drumheads being dull, yellowish, the region of the handle and upper portion red, and the right spot faint, smaller, or absent. The pharynx generally red. The tympanum is inflatable, with a rough blowing sound, after which the appearance of the membrana tympani is not essentially changed. In very rare cases only the affection rises beyond this condition of a mild catarrhal otitis media, developing into purulent inflammation of the drum, with perforation of the drumhead and otorrhœa, which ceases in one or several weeks. These symptoms on the part of the middle ear are, however, of subordinate significance when compared with those furnished by the *inner ear*. In some cases patients at first find sounds around them—for instance, the song of a canary-bird—intolerably harsh, but very soon the hearing-power will diminish, and in nearly all cases be totally and permanently destroyed. When the patients retain their consciousness, impairment of hearing may be noticed as early as the second day of the disease, increasing day by day to total deafness in a week or two. In the majority of cases the deafness is only discovered when the patient awakes from his stupor; and may then be total or partial, increasing, in the latter instance, to total deafness very soon. I find, however, in my notes some cases in which hearing was still present some weeks after the disappearance of the severe symptoms of the acute disease, and was lost during the recovery. In all cases the deafness was bilateral, and, with two exceptions of faint perception of sound, complete. Among the twenty-nine cases of total deafness there was only one who seemed to give some evidence of hearing afterward. The treatment consisted in leeches behind and before the ear, blisters, tincture of iodine, ung. tart. stibiat. behind the ears, and the use of the galvanic current. I have seen no good results from this treatment, nor have I heard of a better one to substitute it.

In conclusion I may say a few words concerning the age of the patients, the mortality of the epidemic, and the nature of the ear-disease. The majority of the patients were under 10 years; above that age there were one of 10, 12, 13, 14, 16 years respectively, and two of 18 years. The epidemic does not seem to show a great rate of mortality; Dr. Ch. F. Rodenstein, of Westchester, N. Y., states only 10 per cent., while other epidemics showed a death-rate from 30 to 75 per cent. The nature of the ear-disease is, in all probability, a purulent inflammation of the labyrinth, by which the membranes of the inner ear are destroyed in a similar way as the membranes of the eye by the purulent choroiditis. Heller and Lucae have corroborated this by three post-mortem examinations. No disease of the middle ear could annihilate the hearing so completely that no no sounds whatever, not even a tuning-fork vibrating on the cranial bones, is perceived. The deafness cannot be the consequence of a destruction of the acoustic nerve within the brain, or of the center of audition; for it would be unexplainable why the adjacent facial or other cranial nerves are not, sometimes at least, found destroyed too. An additional proof is furnished by Dr. Guening, who electrized the greater number of these patients according to Brenner's method, and obtained the normal reaction of the acoustic nerve—a fact which excludes the destruction of the nerve and center of audition.

In communicating these observations to the profession, may I ask the favor to be invited, if not inconvenient, to assist in post-mortem examinations of lethal cases of cerebro-spinal meningitis?—*Med. Record.*

### PLEURITIC EFFUSIONS IN CHILDREN.

Dr. Rehn (*Jah. f. Kinderheilk*, 1872), says that in ten years' practice in Hanau he noticed eight cases of pleuritic effusion in children. Of these there were seven boys and one girl; the eldest child was about five years, the youngest eleven months. In two children, whom he remarked at the commencement of the disease, he noticed a pneumonic attack as preliminary. With regard to the rest of the cases, he is not able to fix on any etiology, since they came to him suffering already from effusion. In none of said children was there any other affection noticeable. The effusions in two cases were, in all probability, serous; one was probably purulent, and the rest were clearly purulent. Six effusions were on the left side, one on the right; in one case, which he treated about nine years before, he does not quite remember the side. In two children, where the effusion according to his opinion was serous, resorption took place in two or three months; in one child (whilst he was absent on a journey) a spontaneous opening of the empyema took place both outwardly and inwardly, and the child got well. Five children were operated on by him in company with a colleague, four by an incision with or without previous puncture, and one by puncture alone. Three of those operated on died, two got well; one with chronic purulent exudation after six months' treatment, the other, a child of one and three-quarters years old, with running of pus, which continued for fourteen days and then got well after (he will not say on account of) once puncturing the chest. It lies not in the intention of the author to publish observations which have already often been published, he will content himself to relate shortly the operative processes in order to make some general remarks on these.

The first case of empyema occurred in a child of eleven months, which had been suffering in the country for some six weeks from fever, cough, dyspnœa, etc. The child was found to be asphyxiated, with a notable left-sided effusion ; the general nutrition was good. Puncture was at once undertaken, and about half a chopin of inodorous pus was withdrawn; on the next day the wound made by the trocar.was enlarged by the bistoury. After apparent improvement there supervened a deterioration after the fifth day, evinced by elevation of temperature, cyanosis, increase of cough, want of nutrition, and loss of sleep, without any lessening of the effusion. On the other hand, there was over the posterior surface of the right side and thence towards the left, a sometimes greater, sometimes less, extended *rale* of coarser or finer description. The child died on the eighth day, and the *post mortem* examination showed the heart pushed over to the left side with centers of lobular pneumonia on the back part of the lung on the right side with bronchitis. In the left plural sac about half a chopin of greenish fluid; the pleura was much thickened, as also the aortal pleura; the left lung pushed up against the vertabræ, the upper lobe containing secretion. The bronchi hyperæmic.

In the second case the author was called into consultation to a boy of three and three-quarters years of age. There was extensive empyema on the left side with pressure of the heart toward the right side of the chest, the spleen was depressed. Puncture was made use of on 1st December, 1868, and about one and a half chopins of pus escaped.

On the 8th it was renewed, the wound was made larger and a canula left in. After a decided amelioration, about the 11th day the wound became vividly red. This turned to diphtheritic appearance, with hardness of the edges, with erysipelas and death. On *post mortem* examination, the heart was found dislocated much to the right, the right lung emphysematous, the left lung pushed backwards and upwards, and fixed in its place by growing together of both pleural envelopes. The upper lobe of the left lung was for the most part œdematous, whilst the lower lobe was soft and free from air.

In the third case a strong child of one year old became ill on 23d May, 1869, with pleurisy on the right side in the upper lobe and posterior aspect, and an exudative pleurisy quickly supervened. Apparently this was quite absorbed by the end of September, but the child again fell sick, and in October the whole of the right side of the chest was filled with effusion, which pushed down the liver, etc. Puncture with a trocar let out a great amount of pus, the wound was afterwards widened, a canula inserted, or a catheter to get away the pus. After great change in the symptoms, the making of pus ceased in about seven months, but there still remained a small fistula out of which a purulent secretion exuded. The child became, however, very strong and healthy.

In the fourth case of empyema, a boy of one year and eleven months, was attacked in October, 1870. The child was much depressed in nutrition and looked very pitiable. The effusion was also on the left side, measurements giving under the axilla on the left side 25 centimetres, and on the right only 23 centimetres. Over the nipple 26 centimetres at left, and 23 at right side, and at the level of the ziphoid cartilage, 27 centimetres on the left side, and only 24 on the right. The heart and spleen were both pushed out of place. The operation was by incision, and tedious treatment of the child was required by daily washing out the pus by the catheter, then using drainage tubes, etc. The child got much better until the middle of January, 1871, when headache, vomiting, sleepiness, and all the symptoms of baseless meningitis appeared, and the child died on January 23d. The *post mortem* gave exactly the same symptoms as in Case 1.

In the fifth case, a child one and three-quarters years old, had on September 23d, fever, dyspnœa, and cough. On October 5th, here the effusion was again on the left, Dr. Rehn determined on early operation, and on October 7th, made a puncture, drawing off half a chopin of thick, greenish fluid. The result was an astonishing recovery of the patient. The respirations sank from 52 to 32 on the third day; but a new effusion appeared, which was treated by digitalis, iodide of potassium, and iron, and gave way in about three or four weeks. Most cases seem to have occurred in boys, and most were under two years of age. The effusion was almost always on the left side and purulent. The most important fact to be noticed, is the great and pressing utility of early operative treatment in pleuritic effusions, not only in purulent cases, but in acute and serous effusions. The operation is so slight as to be destitute of danger and should be made use of.—*The Doctor.*

PLEURITIC EFFUSIONS TREATED BY DRAINAGE.—In *La France Medicale*, 6th July, there are several cases mentioned, where the drainage of Dr. Cazenave has been successfully employed. In one case (Observation IX.) a patient entered the Saint Louis Hospital in May, 1864, under Dr. Cazenave, with double pleurisy. There was persistent effusion on the left side, with imminent asphyxia; thoracentesis was employed, and a quantity of pus withdrawn. There was persistence of the symptoms until August, 1865. In September, 1865, a new puncture was made, giving issue to much pus. In about fifteen days afterwards a fistula appeared at the opening of the puncture, with diarrhœa and sweating, and there were fever and nocturnal exacerbation. Drainage tubes were then used. The diarrhœa, sweats, and fever disappeared, the health was better, and the appetite returned. The patient kept the tube in all the winter, *i.e.*, about seven months, and at the end of February, 1866, no more pus issued from the chest. This patient was examined anew with particular care by Dr. Ranvier. This patient was treated by drainage also, in the service of Professor Gosselin, who sent him to Vincennes almost cured and so much better, that after wearing drainage tubes for more than seven months, he was found to be quite well. "This observation," says Dr. Manny, "proves the cessation of hectic symptoms whenever pus can flow continuously; but the presence of such symptoms of putridity whenever the liquid sojurns for any length of time in the pleural cavity; the innocence of the drainage tube; the dilation of the lung which has been compressed for some time; and the marked disappearance of narrowing of the thoracic cavity."

In another case of left purulent effusion, a spontaneous opening took place in August, 1864, and several quarts of pus issued. The patient had fistulous openings on the chest walls with interminable suppuration and gradual emaciation. Up to 25th August, 1866, there were constant symptoms resulting from retention of pus in the cavity, loss of appetite, vomiting, and disturbed respiration. The patient was forced to sleep in a sitting posture, had diarrhœa and fever with shivering fits. M. Gosselin on the 10th September, introduced a draining tube by means of a curved trocar and a large quantity of pus came away. In November, 1866, the patient was getting better and better. There was scarcely any suppuration, there was return of vesicular breathing, and the lung was dilating.—*The Doctor.*

INDIA-RUBBER CLOTH IN DISEASES OF THE SKIN.—Since 1868 (*Journal de Med. et de Chir. Pratiques*), M. Hardy, of Paris, has been employing india-rubber cloth in place of poultices or local baths. He employs pieces of cotton, covered with a layer of caoutchouc, and forming an impermeable tissue. This is only applicable to the extremities and to the head; and for the latter region he makes use of vulcanized india-rubber caps. After a certain time the part enveloped becomes not disagreeably warm, and then an abundant sweating takes place, under the influence of which the crusts, and the squames which cover the skin are removed, the epidermis spreads over the ulcers, and the skin becomes softened. The results obtained are similar to those obtained by poultices, but preferable for many reasons. Daily experience of the Hospital St. Louis shows a great rapidity in the modification of the skin; two or three days of application suffice completely to cleanse the scalp when covered with abundant scales of eczema, etc. After forty-eight hours application of the india-rubber cloth upon hands attacked with chronic eczema, with fissures and cracks in all directions, the wound becomes cicatrised, and the skin recovers its suppleness. This treatment is especially of great value in eczema in the second period of the disease. The crusts are removed, the plastic secretions disappear, and a thin epidermis, glistening and smooth, gradually covers the diseased part, and the eczema rapidly enters the third period. Itching is rarely relieved by this method. In ecthyma the pustules open, and the cure proceeds very quickly, thanks to the envelope. It must be noticed that the cloth must be covered with india-rubber, for the trial made with oiled silk did not succeed. The limbs should be completely enveloped; but the india-rubber cloth ought only to be tied on at the ends—between the ligature it should form a large sleeve. It should be kept on for a considerable time, and the patient should lie in bed. This method is also employed to make the scales of the psoriasis fall, or to restore the suppleness to the skin in chronic lichen.—*Ibid.*

VITAL TEMPERATURE.—Dr. Jacobson, of Konigsberg, reports in Virchow's *Archives* (vol. li, 2d part) a series of experiments upon animals relative to the temperatures of certain viscera, as measured by thermo-electricity. He has found, contrary to the assertion of Cl. Bernard, that the blood is warmer in the right than in the left heart; but has verified the assertion of the same author, that the temperature of the liver is superior to that of the axilla and the rectum. He has also observed that the temperature of the skin and muscles, when exceedingly inflamed, never attain a temperature equal to that of the interior of the body, or to that of the upper part of the vagina or rectum. By experiment on animals, in which pleuritis is caused, they verified the assertion of J. Hunter, that "blood inflammation can never attain a higher temperature than that found at the source of the circulation."—*Med. Record.*

Indigestion and its Management.—Dr. Bradford S. Thompson, N. Y. (*Am. Practitioner*, July, 1872), in his paper on this subject, alludes to a case under his observation where everything appeared to the patient double; in another, every object seemed inverted; and in another, total blindness came on, which continued for twenty-one hours. The latter patient, a conch-woman, of Key West, Florida, aged 47, was in the habit of eating prodigiously of a salad made from the indigestible conch, which abounds in that latitude.

In the indigestion of childhood the use of Boudault's pepsin wine can be highly recommended. This is prepared from pure pepsin, according to the formula of Dr. Corvisart, and is very palatable. Each dose possesses fully the digestive power of fifteen grains of the powder. This preparation, he states with much confidence, is superior to all other preparations of pepsin in use. It should be given immediately before a meal.

In regulating the diet, the author would impress upon the patient the necessity of observing the subjoined rules: 1st. Enjoin frequent and regular eating in the majority of cases. It was a remark of Sir William Temple, "that the stomach was like a schoolboy; if idle, always in mischief." The deduction drawn from this is to keep the stomach moderately employed. 2d. Let the diet be simple, always consisting exclusively of one article. 3d. Drink little or nothing while eating. 4th. Exercise should not be permitted directly after eating. In many cases a voracious appetite attends this affection; but in the majority of cases there is very little inclination to eat; and under these circumstances it will not be amiss to attend to the following particulars for the purpose of exciting the appetite: Do not let the patient know what he is to eat. The food should always be cold. When hot, the odor will often destroy the appetite. The dishes should always be small; for nothing is more distressing to a patient with a delicate stomach than a large dish of meat placed before him. These circumstances, though apparently trivial in their character, are very important, and deserve recollection.—*Med. Record.*

Blue Light as an Organic Stimulant.—At a recent meeting of the Philadelphia Agricultural Society, General Pleasanton read an interesting paper on the effects of sunlight on plants and animals, when transmitted through blue glass. Geraniums which had become unhealthy recovered their vigor and became more deeply colored when covered with blue glass; and the branches of the same grape-vine showed a remarkable difference in their growth of leaves according as they were or were not covered with blue glass, the leaves on the former having a diameter of six to eight inches, being of a deep-green color and perfectly healthy; while the uncovered branches were only two inches in diameter, and of a pale, sickly yellowish color, indicating a feeble vitality. The paper also mentioned a case of the wife of a Philadelphia physician who had for some time been suffering from a complication of disorders which had baffled the skill of her physicians, and who, on the suggestion of General Pleasanton, tried the following plan: Every other pane of glass in one of the windows of the patient's room was removed and the blue glass substituted, and the patient required to expose her back and spine to the action of the combined blue and white lights for thirty minutes each day, at the same hour. At the commencement of treatment she was unable to sleep or eat, was in a miserable condition, and wasting rapidly. At the end of ten days the pains in her back were less, her hair had commenced growing thickly, and there was a marked improvement in her general condition. In three weeks she was almost entirely well.—*Ibid.*

The Treatment of Pleurisy.—Doctor Quincke, of Berlin, (*Berliner Klin. Woch.*) mentions that he had, under Dr. Fredrichs, the opportunity of treating fifteen cases of effusion in pleurisy by operating. He comes to the following conclusion:—In order to get rid of serous exudations, the most practicable method appears to be puncture with a fine trocar, and withdrawal of the fluid by suction. Even when nothing more than a palliative result is to be expected puncture is often indicated. In exudations of pus, incision, and afterwards daily out-cleansing of the cavity of the pleura is indicated at once, and this should be done early, since a spontaneous absorption is unlikely to take place, and at any rate is very tedious. Even in many cases of purulent pleurisy, which turn into pneumo-thorax, operative procedures are indicated.—*The Doctor.*

Anti-Phlogistics in Myelitis.—Dr. Reignier (*La France Medicale*) gives two or three cases of myelitis successfully treated by him. A man, æt. 45, a peasant proprietor, was greatly addicted to drinking wine. April 20th, he experienced formication in the feet and hands, and was forced to discontinue his work. On May 25th there was total loss of power in his locomotory organs. Tongue furred, pulse full but not accelerated, no dyspnœa or constrictive pain around waist. On percussing along the spine no painful sensation was experienced. Micturition was easy; but constipation obstinate. Intelligence clear. Was this congestion or inflammation? Thinking it might be congestion, thirty leeches were placed along the spine, revulsions were applied to the extremities, drastic purges used, and low diet. The paralysis continued, and intolerable pains shot through the limbs like lightning. He then was treated by revulsions, and ten cauteries were applied along the vertebral column, five on either side, from above, downwards, to the level of the loins. On the 28th May, a month after applying the cautery, there was great amelioration. On the 15th June he could stand, and on the 23d could move without a stick.

A New Theory of Asthma.—In the sputa of asthma, also in the spleen of patients who have died with leukemia, certain colorless, octohedral crystals have been observed, says the editor of the *Pacific Medical Journal*, by European microscopists. Some of the crystals are large enough to be detected with the naked eye. Professor Leyden, of Konigsberg, as appears from an extract from *Virchow's Archives*, in the *Philadelphia Medical Times*, regards them as the cause of the bronchial spasm in asthma. He advises asthmatic patients to inhale a solution of common salt and carbonate of soda, for the purpose of dissolving the crystals and preventing their formation.

Lady Medical Students.—It appears that out of four hundred students attending the University at Zurich, no less than eighty are ladies. Most of them are medical students, and, what is strange, most from Russia.—*The Doctor.*

